# A photoacoustic imaging reconstruction method based on directional total variation with adaptive directivity

**DOI:** 10.1186/s12938-017-0366-3

**Published:** 2017-05-30

**Authors:** Jin Wang, Chen Zhang, Yuanyuan Wang

**Affiliations:** 1 Department of Electronic Engineering, Fudan University, Shanghai, 200433 China; 2 Key Laboratory of Medical Imaging Computing and Computer-Assisted Intervention of Shanghai, Shanghai, 200433 China

**Keywords:** Photoacoustic tomography, Image reconstruction, Directional total variation, Directivity adaptive

## Abstract

**Background:**

In photoacoustic tomography (PAT), total variation (TV) based iteration algorithm is reported to have a good performance in PAT image reconstruction. However, classical TV based algorithm fails to preserve the edges and texture details of the image because it is not sensitive to the direction of the image. Therefore, it is of great significance to develop a new PAT reconstruction algorithm to effectively solve the drawback of TV.

**Methods:**

In this paper, a directional total variation with adaptive directivity (DDTV) model-based PAT image reconstruction algorithm, which weightedly sums the image gradients based on the spatially varying directivity pattern of the image is proposed to overcome the shortcomings of TV. The orientation field of the image is adaptively estimated through a gradient-based approach. The image gradients are weighted at every pixel based on both its anisotropic direction and another parameter, which evaluates the estimated orientation field reliability. An efficient algorithm is derived to solve the iteration problem associated with DDTV and possessing directivity of the image adaptively updated for each iteration step.

**Results and conclusion:**

Several texture images with various directivity patterns are chosen as the phantoms for the numerical simulations. The 180-, 90- and 30-view circular scans are conducted. Results obtained show that the DDTV-based PAT reconstructed algorithm outperforms the filtered back-projection method (FBP) and TV algorithms in the quality of reconstructed images with the peak signal-to-noise rations (PSNR) exceeding those of TV and FBP by about 10 and 18 dB, respectively, for all cases. The Shepp–Logan phantom is studied with further discussion of multimode scanning, convergence speed, robustness and universality aspects. In-vitro experiments are performed for both the sparse-view circular scanning and linear scanning. The results further prove the effectiveness of the DDTV, which shows better results than that of the TV with sharper image edges and clearer texture details. Both numerical simulation and in vitro experiments confirm that the DDTV provides a significant quality improvement of PAT reconstructed images for various directivity patterns.

## Background

Photoacoustic tomography (PAT), also referred to as optoacoustic tomography, is an emerging biomedical imaging modality. It combines a high contrast of the optical imaging with a good resolution of ultrasound one [1] and possesses a noninvasive feature [2], which unique advantages over other traditional imaging techniques help it to find wide applications in many aspects of biomedical fields [3–7], such as small animal imaging [8], tumor detection [9], vessel imaging [10], functional imaging [11], and molecular imaging [12]. In the computed-tomographic PAT, which is mainly considered in this study, the laser pulse is usually used to irradiate the biomedical tissue. The tissue absorbs the light and then sends out ultrasound waves. This kind of phenomenon is called photoacoustic effect [1, 2]. A scanning ultrasound transducer or a transducer array is used to detect the generated ultrasound signals around the tissue. The detected signals are then utilized to reconstruct photoacoustic images, which reflect the light absorption of the tissue via a certain algorithm. Therefore, the reconstruction algorithm plays a significant role in the PAT.

Many efforts have been made, in order to find an accurate and efficient photoacoustic image reconstruction method. In 1995, Kruger et al. [6] realized the image reconstruction by utilizing the inverse Radon transform, which is considered a pioneer PAT reconstruction algorithm. After that, they also proposed the inversion of the spherical mean Radon transform method, which was more accurate [13]. The filtered back-projection method (FBP) advanced by Xu et al. [14] was widely applied to PAT, due to its concision and accuracy. Zhang et al. [15, 16] proposed the deconvolution reconstruction algorithm, which had better performances in limited-view sampling and used the heterogeneous speed of sound. There is another type of PAT reconstructed methods called time-reversal, which reconstructs images from a forward-propagation numerical model to generate measured photoacoustic (PA) signals backwards in the time [17–20]. Xu et al. proposed a time-reversal-based reconstructed method for three-dimensional broadband diffraction tomography [17] and applied it to PAT [18]. Bradley et al. [19] used the time-reversal method to compensate for acoustic absorption in the photoacoustic tomography. Cox et al. [20] found the artifact trapping phenomenon in the time-reversal PAT reconstruction and proposed some methods to mitigate these artifacts. Besides the above reconstruction methods, another kind of algorithm, called iterative reconstruction method, has been applied to PAT. This kind of algorithm constructs a forward model, which utilizes the relationship between photoacoustic signals and the light absorption deposition to calculate the reconstructed image iteratively under some optimization conditions [21–23]. Thus, this kind of algorithm is also referred to as model-based algorithm. Paltauf et al. [21] advanced an iteration reconstruction algorithm by minimizing the difference between the detected PA signals and the calculated ones from the image. Ma et al. [22] introduced a filtered mean-back projection-iterative reconstruction algorithm to deal with the linear-array detection in practical application. To accelerate the speed of the iterative reconstruction method, Dean-Ben et al. [24] proposed an angular image discretization model-based reconstruction method. Rosenthal et al. [25] used wavelet packets to considerably reduce the computational cost for the model-based algorithm. In order to improve the performance of the model-based algorithm under the circumstance of sparse sampling, the compressed sensing (CS) theory has been employed [26, 27]. The total variation (TV) is an important sparsity regularizer in the image denoising and CS image reconstruction [28]. It utilizes the sparsity of the natural image gradient to measure the variations in an image [29]. In PAT, Wang et al. [30] presented an adaptive steepest-decent-projection onto convex sets (ASD-POCS) method, which involved the TV in the iteration. Zhang et al. [31] proposed a gradient descent algorithm based on the TV, which provided better results, especially under the condition of sparse sampling. Arridge et al. [32] used the TV regularization enhanced by Bregman iterations to solve the PAT sub-sampling problems, which achieved both increased acquisition speed and good spatial resolution. However, the TV is only a measure of local changes in images, which is not related to the directions of images. It is reported that the TV-based method tends to produce over-smoothed image edges and texture details [33–35]. Thus, the isotropic TV-based PAT reconstructed methods are more applicable to images, which are piecewise-smooth and have no dominant direction, but their operation is deteriorated when applied to images with directional textures. At present, there is no PAT reconstruction algorithm to effectively solve the TV drawback.

In this study, we propose a directivity adaptive direction total variation (DDTV) based PAT image reconstruction algorithm for effective minimization of the TV deficiencies. The spatially varying directivity pattern of the image is firstly estimated. Then the image gradients are weighted by the calculated orientation field of the image. The DDTV is calculated by summing the norm of the weighted gradients. The gradient-based approach [36, 37] is used to efficiently estimate the orientation field of the image. Meanwhile, we also calculate the reliability of the estimated orientation field *C*_*k*_, which is used to weight the TV in the chosen direction [38]. This makes the DDTV applicable to any kind of images with various directional patterns. Moreover, we also derive an efficient algorithm to solve the iteration problem associated with the DDTV having the directivity of the image adaptively updated for each iteration step.

Finally, the DDTV algorithm is verified through the numerical simulation and in vitro experiments and compared with the FBP and TV. Results obtained show that the DDTV surpasses those two algorithms both in visual quality and quantitative indices. The proposed algorithm demonstrates its superiority, especially for texture images with obvious directivities, where the image edge is preserved better and the texture details are more distinct. In addition, we also compare the peak signal-to-noise rations (PSNR), convergence speed, and robustness of the DDTV-based method with those of FBP and TV algorithms.

Arridge et al. [32] also used the TV as the regularizer to solve the PAT reconstruction problem, but their algorithm is quite different from the one proposed in this study, since researchers [32] mainly study the PAT sub-sampling problems, while our work is mainly focused on the PAT reconstruction problem. Moreover, the TV regularizer used in [32] is the classical TV, which is isotropic and has no relationship with various directivity patterns of images. In this paper, we propose the novel DDTV, which is sensitive to the directions of images. Thus, the edges and texture details of images can be preserved better. Besides, Tick et al. [39] proposed a Bayesian approach-based PAT, which estimates the initial pressure distribution accurately with the uncertainty quantification. In this study, we mainly use a novel DDTV as the sparsity regularizer to solve the PAT reconstruction problem. The Bayesian approach is a probability estimation method, which treats parameters as random variables, and the solution is based on the knowledge of prior information. The DDTV-based algorithm, which is solved iteratively, also needs the directional information of images. However, the directivity of images is estimated and updated for each iteration step with no prior information of the image.

The adaptive directional total-variation (ADTV) model described in [38, 40] is used in latent fingerprint segmentation with the purpose of decomposing an input image into two layers: cartoon and texture. The DDTV model proposed in this study is applied to the model-based PAT reconstruction problem as a regularizer in the optimization. Although both DDTV and ADTV models use the same orientation field estimation method and weight the normal TV via spatially varying directions of images, there exist major differences between them. The orientation field estimation of both methods implies calculation of two parameters: orientation *θ*_*k*_, and reliability of the estimated orientation field *C*_*k*_. In the ADTV, the orientation vector *α*_*k*_ is computed by multiplying the orientation field (−cos*θ*_*k*_, sin*θ*_*k*_) and *C*_*k*_. The ADTV is obtained by the dot product of the gradient of cartoon layer *u* and orientation vector *α*_*k*_. Thus, for the regions with strong orientation patterns, where the value of *C*_*k*_ is large, the textures of this orientation in cartoon layer *u* are fully depressed, while in the texture layer *v* they are fully captured. As for the isotropic regions, where *C*_*k*_ approaches zero, *α*_*k*_ becomes a zero vector, so that the ADTV term becomes neglectable, while *u* depends only on the fidelity term. However, the DDTV in this study is obtained by replacing the unit ball of the L_2_ norm in the TV, which is directionless with spatially varying ellipses having minor axis unit length and major axis length *α* exceeding unity and oriented along the direction *θ*_*k*_. The major axis length *α*_*k*_ of the eclipse is calculated via *C*_*k*_. At regions with strong orientation patterns, where *α*_*k*_ becomes maximum, the TV in that direction is amplified to the largest, while the TV in other directions are weighted depending on the axes of eclipse in these directions. This is different from the ADTV, where the TV in other directions are fully depressed for a strong orientation pattern. As for isotropic regions, where *α*_*k*_ reaches unity, the eclipse turns into a unit circle and the DDTV is reduced to the normal TV. This is also different from the ADTV, wherein the ADTV term is neglectable and the fidelity term becomes dominant. The method proposed in this study is coined DDTV as an abbreviation for “directional total variation with adaptive directivity” to distinguish it from ADTV. Berkels et al. [41] mainly dealt with the cartoon extraction from aerial images, which are mainly characterized by rectangular geometries with varying orientation. They only use the rotation angles to calculate the orthogonal matrix and its dot product with the cartoon part gradients, which is quite different from DDTV that uses both anisotropic directions and the estimated orientation field reliability to weight the image gradients.

The rest of this paper is organized as follows. The first section is “Background”, the second one is the derivation and the framework of the algorithm, “Numerical simulations” describes the numerical simulation results, while the experimental results are presented in “Experimental results”. The last section presents the discussion and conclusion.

## Theory and method

### Model-based photoacoustic theory

When irradiated by a laser pulse, the biological tissue absorbs the laser energy and generates ultrasound signals according to the photoacoustic effect. The photoacoustic signals and the laser absorption deposition obey the following relationship [1]:

1 $$\nabla^{2} p({\mathbf{r}},t) - \frac{1}{{c^{2} }}\frac{{\partial^{2} }}{\partial t}p({\mathbf{r}},t) = - \frac{\beta }{{C_{p} }}\frac{\partial }{\partial t}H({\mathbf{r}},t),$$

where *p*(**r**, *t*) is the acoustical pressure measured at the time t and the position **r**, *c* is the speed of sound, *C*_*p*_ is the specific heat, *β* is the isobaric expansion coefficient. *H*(**r**, *t*) is a heating function which can be written as:

2 $$H ({\mathbf{r}} ,t ) = A ({\mathbf{r}} )I (t ) ,$$

where *A*(**r**) is the spatial optical absorption distribution of the tissue and *I*(*t*) is the temporal laser pulse function.

In this paper, we only consider the two-dimension PAT and assume spatially uniform laser irradiation with the laser pulse approximating Dirac’s delta function. A transducer scans the photoacoustic signals at several positions. Then (1) can be solved by a Green’s function and the acoustic pressure detected by the transducer at position **r**_0_ can be written as [42]:

3 $$p({\mathbf{r}}_{0} ,t) = \frac{\beta }{{4\pi C_{p} }}\frac{\partial }{\partial t}\mathop{{\int\!\!\!\!\!\int}\mkern-21mu \bigcirc}\nolimits_{{\left| {{\mathbf{r}} - {\mathbf{r}}_{0} } \right| = ct}} {\frac{{A({\mathbf{r}})}}{t}d^{2} {\mathbf{r}}} .$$

The analytical reconstruction algorithms are mainly focused on the inverse problem the solutions of (3) to obtain the optical absorption distribution of the tissue *A*(**r**). However, the model-based PAT makes use of (3) to establish a forward model.

Define a new variable *g* as:

4 $$g({\mathbf{r}}_{0} ,t) = \frac{{4\pi C_{p} t}}{\beta }\int_{0}^{t} {p({\mathbf{r}}_{0} ,t)} dt.$$

Then, integrating both side of (3), the following equation is derived:

5 $$g({\mathbf{r}}_{0} ,t) = \mathop{{\int\!\!\!\!\!\int}\mkern-21mu \bigcirc}\nolimits_{{\left| {{\mathbf{r}}_{0} - {\mathbf{r}}} \right| = ct}} {A({\mathbf{r}})} d{\mathbf{r}}.$$

One can see that the right side of (5) is the line integral of *A*(**r**) with the path of an arc centered at **r**_0_ and the radius of *ct*.

In practical experiments, where the detected signals as well as the photoacoustic image tend to be discretized, *g* for the *l*th detection point is discretized to a vector **g**_*l*_ with the length of S, while image *A* is discretized to a matrix **A** with the size of *N*_*x*_×*N*_*y*_. As it follows from (5), in the discrete form, each element in vector **g**_*l*_ can be expressed via the weighted sum of the elements in matrix **A**. The size of the weight matrix corresponding to the *h*th element of **g**_*l*_ is *N*_*x*_×*N*_*y*_. Then matrix **A** and weight matrix are reshaped to the column vector **A**′ and ${\mathbf{W}}_{l}^{h}$, which have the same length of N_x_·N_y_. Thus, the *h*th element in **g**_*l*_ can be expressed via the dot product of ${\mathbf{W}}_{l}^{h}$ and **A**′. Arranging these weight vectors ${\mathbf{W}}_{l}^{h}$ in order, the weight matrix **W**_*l*_ corresponding to **g**_*l*_ with the size of N_x_·N_y_×S can be constructed. Therefore, for *l*th detection point, **g**_*l*_ can be expressed as the dot product of **W**_*l*_ and **A**′ [21]:

6 $${\mathbf{g}}_{l} = {\mathbf{W}}_{l}^{{\mathbf{T}}} \cdot {\mathbf{A^{\prime}}},\quad l = 1,2,3, \ldots ,M,$$

where **M** is the number of the detection points.

The *m*th element in the weight vector ${\mathbf{W}}_{l}^{h}$ can be written as [31]:

7 $$\begin{array}{*{20}c} {W_{l}^{h,m} = \left\{ {\begin{array}{*{20}c} {1 - \left| {\frac{{t_{h} }}{\Delta t} - \frac{{\left| {{\mathbf{r}}_{l} - {\mathbf{r}}_{m} } \right|}}{c\Delta t}} \right|} & {when \, \left| {\frac{{t_{h} }}{\Delta t} - \frac{{\left| {{\mathbf{r}}_{l} - {\mathbf{r}}_{m} } \right|}}{c\Delta t}} \right| < 1} \\ 0 & {else} \\ \end{array} ,} \right.} & {} \\ \end{array}$$

where **r**_*l*_ refers to the position of *l*th sampling point and **r**_*m*_ refers to the position vector for the *m*th element in the matrix **A**, *t*_*h*_ is the time for *h*th measurement at *l*th detection point, and Δ*t* is the step size of the discretized time. The weight matrices are determined via the integral path. The elements inside/outside of the integral arc are set to 1 and 0, respectively. However, in practical discretized systems, there are discrete intervals for each discretized variable, so the integral arc may not fully coincide with the discretized points. To reduce this error, we calculate the absolute value $\left| {\frac{{t_{s} }}{\Delta t} - \frac{{\left| {{\mathbf{r}}_{l} - {\mathbf{r}}_{m} } \right|}}{c\Delta t}} \right|$, which is the error between the position of the *m*th element in the image and the accurate position of the integral arc. When this value exceeds unity, which means that the error exceeds one discrete interval, then this element is not located on the integral arc. When the value is less than one (discrete interval), the weight is from 0 to 1 based on the error calculated by the absolute value. The lager the error, the less the weight, and vice versa. For practical discrete system, *g* is readily calculated by accumulating values of *p*. The integral path is determined by the detection points’ and image positions, i.e., the PAT sample pattern. When the latter changes, for example, by shifting from circular scanning to straight-line one, the weight matrix **W** has to be adjusted, according to the sample pattern, making applicable the forward model from **A** to **g** in (6). A simple replacement of *g* by *p* makes the forward model quite straightforward, transparent, and flexible.

### Directivity adaptive directional total variation (DDTV)

The traditional TV, which measures oscillation in an image, is described by the following equation for a discrete image:

8 $$TV({\mathbf{\rm A}}) = \sum\limits_{i,j} {\left[ {\left( {A_{i,j} - A_{i - 1,j} } \right)^{2} + \left( {A_{i,j} - A_{i,j - 1} } \right)^{2} } \right]^{1/2} } ,$$

where *A*_ij_ is the pixel value of the image at the coordinate (*i*,*j*).

The alternative way to express the TV [43] is:

9 $$TV({\mathbf{A}}) = \sum\limits_{i,j} {\left\| {\nabla A_{i,j} } \right\|_{2} = } \sum\limits_{i,j} {\mathop {\sup }\limits_{{p \in {\rm B}_{2} }} \left\langle {\nabla A_{i,j} ,p} \right\rangle } ,$$

where *B*_2_ is the unit ball of the L_2_ norm, and *sup* is the supremum function. It is obvious that the TV is isotropic, because *B*_2_ is directionless. Here $\nabla$ is a linear operator defined as:

10 $$\begin{aligned} \nabla A_{i,j}& = \left( {G_{1} ,G_{2} } \right) \\ &= \left( {A_{i,j} - A_{i - 1,j} ,A_{i,j} - A_{i,j - 1} } \right), \\ \end{aligned}$$

where *G*_1_ and *G*_2_ are horizontal and vertical gradients of *A*_*ij*_ respectively.

The replacement of *B*_2_ by an ellipse *E*_*α*,*θ*_, which has a unit length minor axis and a major axis length *α* greater than 1 and is oriented along the direction *θ*, makes the TV more sensitive to changes at the certain direction, whereas the directivity intensity of that direction is measured by *α* [43]. Thus, the directional TV (DTV) can be written as:

11 $$DTV_{\alpha ,\theta } = \sum\limits_{i,j} {\mathop {\sup }\limits_{{p \in E_{\alpha ,\theta } }} \left\langle {\nabla A_{i,j} ,p} \right\rangle } .$$

In [43], only one kind of ellipse is chosen, which has a single direction *θ* and *α*. This approach is not universal, since, in most cases, images may have heterogeneous directivity patterns. To overcome this problem, we propose the DDTV with spatially varying *θ* and *α*.

The same approach as in [38] is adopted to calculate the spatially different directivity patterns of the image. In order to reduce the calculation efforts, the image is firstly subdivided into blocks of the same size. Then the coarse orientation field for each block is calculated by using the gradient-based approach [36, 37]:

12 $$O_{k} = \frac{1}{2}\tan^{ - 1} \frac{{\sum\nolimits_{{\mathbf{L}}} {2G_{1} G_{2} } }}{{\sum\nolimits_{{\mathbf{L}}} {\left( {G_{1}^{2} - G_{2}^{2} } \right)} }} + \frac{\pi }{2},$$

where L is a number of pixels within the block.

To improve the estimation accuracy, *O*_k_ is further smoothened by the Gaussian smoothing kernel *G*_δ_ [38]:

13 $$\theta_{k} = \frac{1}{2}\tan^{ - 1} \left\{ {\frac{{G_{\sigma } \times \sin \left( {2 \cdot O_{k} } \right)}}{{G_{\sigma } \times \cos \left( {2 \cdot O_{k} } \right)}}} \right\}.$$

Simultaneously, another parameter *C*_*k*_ is calculated, which evaluates the dependability of the estimated direction for each block [38]:

14 $$C_{k} = \frac{{\left( {\sum\nolimits_{{\mathbf{L}}} {\left( {G_{1}^{2} - G_{2}^{2} } \right)} } \right)^{2} + 4\left( {\sum\nolimits_{{\mathbf{L}}} {G_{1} G_{2} } } \right)^{2} }}{{\left( {\sum\nolimits_{{\mathbf{L}}} {\left( {G_{1}^{2} + G_{2}^{2} } \right)} } \right)^{2} }},$$

where *C*_*k*_ represents the directivity intensity of *k*th block within the range of [0,1]. For directionless or isotropic regions, *C*_*k*_ equals to 0, while for strongly oriented regions, *C*_*k*_ approaches 1 [38]. Values *θ*_*k*_, and *C*_*k*_ are used as the orientation field parameters for all pixels within the block under study.

After completing the calculation, two parameters *θ*_*i,j*_ and *C*_*i,j*_ are obtained for each pixel. Here, the orientation field parameter *θ*_*i,j*_ is used as the spatially varying *θ* of the ellipse. The major axis length *α* is defined as:

15 $$\alpha_{i,j} = \left( {\alpha_{m} - 1} \right) \times C_{i,j} + 1,$$

where *α*_*m*_ is the defined maximum major axis length. For the directionless pattern, *C*_*i,j*_ equals to 0 and *α*_*i,j*_ equals to 1. The ellipse degrades to a circle, which implies that the DTV turns into the TV for that pixel. For the strongest directivity pattern, *C*_*i,j*_ equals to 1 and *α*_*i,j*_ reaches its maximum value *α*_*m*_.

Thus, DDTV can be described as:

16 $$DDTV_{{\alpha_{i,j} ,\theta_{i,j} }} \left( {\mathbf{A}} \right) = \sum\limits_{i,j} {\mathop {\sup }\limits_{{p_{i,j} \in E_{{\alpha_{i,j} ,\theta_{i,j} }} }} \left\langle {\nabla A_{i,j} ,p_{i,j} } \right\rangle } .$$

### DDTV-based PAT image reconstruction algorithm

Consider the following optimization problem, which has to be solved via the DDTV:

17 $${\mathbf{A}}^{ * } = \arg \mathop {\hbox{min} }\limits_{{\mathbf{A}}} \left\| {{\mathbf{W}}^{T} \cdot {\mathbf{A^{\prime}}} - g} \right\|_{2}^{2} + \lambda DDTV_{{\alpha_{i,j} ,\theta_{i,j} }} \left( {\mathbf{A}} \right),$$

where A* is the reconstructed image. *λ* is the parameter corresponding to the weight of DDTV value in the optimization.

For each iteration step, the orientation field of the image is firstly estimated by the updated reconstructed image through the method described in “Directivity adaptive directional total variation (DDTV)”. For each pixel (*i*,*j*), the rotation matrices *R*_*θij*_ and scaling matrices *Λ*_*αij*_ are controlled by *θ*_*i,j*_ and *α*_*i,j*_ [43]:

18 $$R_{\theta ij} = \left[ {\begin{array}{*{20}c} {\cos \theta_{ij} } & { - \sin \theta_{ij} } \\ {\sin \theta_{ij} } & {\cos \theta_{ij} } \\ \end{array} } \right],\,\varLambda_{\alpha ij} = \left[ {\begin{array}{*{20}c} {\alpha_{ij} } & 0 \\ 0 & 1 \\ \end{array} } \right].$$

After each iteration, the reconstructed image is updated and the orientation field of image is reestimated and updated at the start of the next iteration. Thus, a full DTV-regularized inverse problem is solved for each iteration, and then the orientation field is updated.

After that, the problem is solved by minimizing the DDTV via the algorithm proposed in [44, 45]. Define $\varGamma_{ij} \,=\, \mathop {\text{argmin}}\nolimits_{{\varGamma_{ij} \in B_{2} }} \left\| {Grad({\mathbf{A}}_{{\mathbf{s}}} )_{ij} - \nabla^{T} R_{{\theta_{ij} }} \varLambda_{{\alpha_{ij} }} \varGamma_{ij} } \right\|$ and Γ_*ij*_ can be solved via the following iteration scheme [44, 45]:

19 $$\varGamma_{ij}^{{\left( {n + 1} \right)^{\prime } }} = \varGamma_{ij}^{\left( n \right)} + \gamma_{ij} {\mathbf{H}}_{ij}^{T} \left( {Grad\left( {{\mathbf{A}}_{\text{s}} } \right)_{ij} - {\mathbf{H}}_{ij} \varGamma_{ij}^{\left( n \right)} } \right),$$

20 $$\varGamma_{ij}^{{\left( {n + 1} \right)}} = \frac{{\varGamma_{ij}^{{\left( {n + 1} \right)^{\prime } }} }}{{\hbox{max} \left\{ {\left\| {\varGamma_{ij}^{{\left( {n + 1} \right)^{\prime } }} } \right\|_{2} ,1} \right\}}},$$

where *γ*_*ij*_ is a coefficient defined as $\gamma_{ij} = \frac{1}{{8\alpha_{ij}^{2} \lambda^{2} }}$, *α*_*ij*_ is the ellipse major axis length for the pixel (*i*,*j*) calculated via (15), **H**_*ij*_ is defined as ${\rm H}_{ij} = \nabla^{T} R_{\theta ij} \varLambda_{\alpha ij}$. Hereinafter **A**_*s*_ is the updated reconstructed image for the *s*th iteration step, and *Grad* is the defined gradient-descent-based updating operator, which also are included in the following equations:

21 $$\Delta {\mathbf{A}}_{s} = - \frac{{\mathbf{W}}}{{\left\| {\mathbf{W}} \right\|}}({\mathbf{W}}^{T} \cdot {\mathbf{A}}_{s}^{\prime } - g),$$

22 $$Grad({\mathbf{A}}_{s} ) = {\mathbf{A}}_{s} + \Delta {\mathbf{A}}_{s} .$$

As it reported in [44, 45], the final results of this iteration can be obtained from Γ_*ij*_:

23 $${\mathbf{A}}_{{{\mathbf{s}} + {\mathbf{1}}}} (i,j) = Grad({\mathbf{A}}_{{\mathbf{s}}} )_{{_{ij} }} - {\rm H}_{ij} \varGamma_{ij} .$$

Briefly, the iteration steps of the DDTV algorithm can be listed as follows:

1. Initialization: input **A**, *α*_*m*_, *λ*.
2. Estimate the orientation fields of **A**_*s*_ via Eqs. (12)–(15). Calculate the rotation matrices *R*_*θij*_ and scaling matrices *Λ*_*αij*_.
3. For *n* = 1 and the defined step number, run the following iteration via Eqs. (1)–(22).
4. Update the reconstructed image **A**_*s*+1_ via Eq. (23).
5. If the terminal condition is not satisfied, return to step (2) and continue the iteration. Otherwise, end the iteration.

In the numerical simulations and experiments of this study, we set iteration number 10 as the terminal condition for all cases. Set initial input **A** to 0 to avoid the input of unnecessary man-made noise caused by the initial guess. The parameters *α*_*m*_ and *λ* are derived through the experiments.

## Numerical simulations

A series of numerical simulations were carried out to validate the proposed DDTV-based algorithm. In order to validate the superiority of the DDTV-based algorithm in the adaptive directional sensitiveness, two kinds of texture images with various directions were chosen as the simulation phantoms. Circular scanning with different sampling points was simulated, and the DDTV results were compared with those of FBP and TV. Then the Shepp–Logan image, which is often adopted to assess the image reconstruction algorithm, was used to verify the effectiveness of the DDTV algorithm quantitatively and qualitatively through the circular, limited-view, and linear scanning options. The PSNR, convergence speed, and robustness of FBP, TV and DDTV algorithms were also analyzed and compared. Finally, several medical images were used to test the universality of the algorithm. The adaptive tunable parameter for lambda. Which was proposed in [31] for the TV-based algorithm, was used in this study. In this case, the initial lambda value was set to 2 for the first iteration and decreased to 0.2, when the iteration number exceeded 10. The iteration time of 10 was set for all cases under study, wherein lambda was relatively large at the beginning of the iteration and decreased as the iterations continued, which provided a good balance of the two parts of the object function. This adaptive tunable parameter proved to be the most effective for the iteration time of 10 [31]. The parameter *λ* for DDTV was set to maximize the PSNRs of the reconstructed results. In fact, *λ* determines the weight of DDTV term in the optimization and its large value implies that the DDTV-term is dominant. This would result in a quicker convergence of the algorithm, but too large value of *λ* will break the balance between the two parts of the objective function. The reconstructed images with a too large *λ* would much differ from the true ones, due to the data fidelity in the reconstruction being sacrificed to the image regularity. Based on this criterion, a moderate value of *λ*, which is neither too large nor too small, is preferred.

The simulations were conducted using the Matlab R2013a software installed on a PC with a 2.40 GHz Intel(R) Xeon(R) CPU and 64 GB memory. The speed of sound in the simulation was assumed to be constant and equal to 1500 m/s.

### Texture image reconstruction

**Fig. 1:**
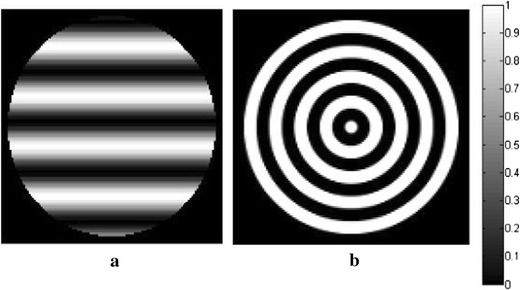
Texture images. **a** Refers to the transverse texture image and **b** refers to the circular texture image

**Fig. 2:**
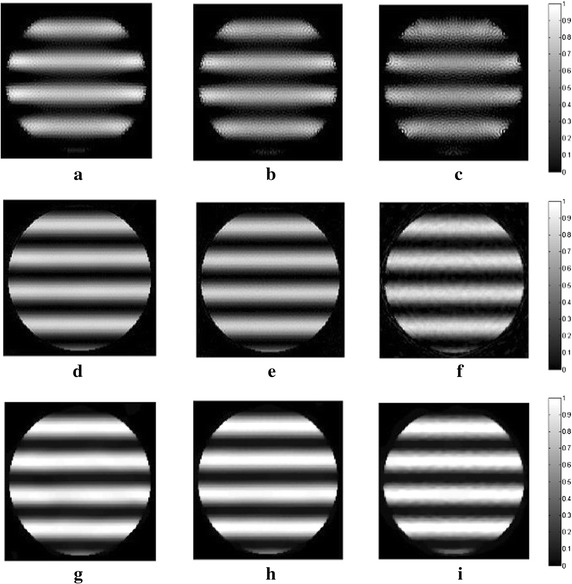
Reconstructed images of the transverse texture from the FBP, the TV and the DDTV algorithm. The first to third *rows* refer to the FBP (**a**–**c**), the TV (**d**–**f**) and the DDTV (**g**–**i**) reconstructed images, respectively. The first to third *columns* refer to 180-view (**a**, **d**, **g**), 90-view (**b**, **e**, **h**) and 30-view (**c**, **f**, **i**) sampling results, respectively

**Fig. 3:**
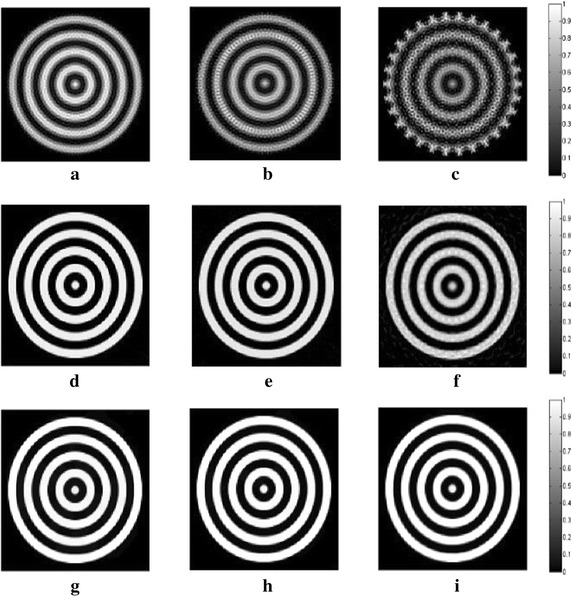
Reconstructed images of the circular texture from the FBP, the TV and the DDTV algorithm. The first to third *rows* refer to the FBP (**a**–**c**), the TV (**d**–**f**) and the DDTV (**g**–**i**) reconstructed images, respectively. The first to third *columns* refer to 180-view (**a**, **d**, **g**), 90-view (**b**, **e**, **h**) and 30-view (**c**, **f**, **i**) sampling results, respectively

Two kinds of texture images are selected as the optical absorption distributions of the phantom: the transverse and circular direction patterns, as is shown in Fig. 1a, b. The image dimensions are 128 pixels × 128 pixels, which correspond to the simulation area of 76.8 mm × 76.8 mm, and the scanning radius is 36 mm. The photoacoustic signals are generated by the modified finite-difference time-domain (FDTD) method [46], which are detected circularly with the sampling points of 180, 90, and 30, respectively, while the angular step size is uniform. The modified FDTD method uses photoacoustic Eq. (3) for the numerically produced simulated data. We use the forward projection model described in “Model-based photoacoustic theory” to reconstruct the image iteratively under DDTV regulation. So the inverse crime can be avoided in our method during the generation of simulated signals. The TV and DDTV iteration numbers are both set to 10, while the parameters *λ* and *α*_*m*_ are set to 1.2 and 10, respectively. In all following simulations, the size of the block is set to 5×5. The TV and DDTV reconstruction results are compared in Figs. 2 and 3, while the FBP results are also depicted as a reference.

The results obtained strongly indicate a mediocre FBP performance: the contrasts of the image patterns are poor and blurring occurs when the sampling points become sparse. The TV fails to preserve the texture details and the edge sharpness of the image, due its insensitivity to the texture direction. Moreover, there are also some artifacts within the textures, which lead to the contrast reduction. It is clear that, in contrast to TV, the DDTV provides a great improvement of the reconstructed image quality for all directive patterns. The image edges and texture details are well preserved even for the sparse-view reconstruction.

**Fig. 4:**
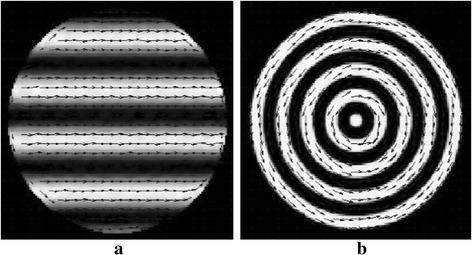
**a**, **b** Refer to the orientation field estimation results for two texture images in the last iteration of 30-view DDTV. The *arrows* stand for the direction for the blocks. The length of the *arrows* indicates the reliability of the estimated orientation field for that position

Figure 4 shows the orientation field estimation results for two images in the last iteration of 30-view DDTV. The direction vectors are multiplied by *C*_*k*_. Here the arrow length reflects the directivity intensity/strength for that block. As seen from Fig. 4, the arrow directions coincide well with the image directions. The results validate the adaptive direction pattern estimation method in the DDTV.

In order to quantitatively measure the image reconstruction algorithms, we also compare the PSNR of the image, which is defined as:

24 $$PSNR = 10 \cdot \log_{10} \left( {\frac{{N_{x} N_{y} \cdot MAXI^{2} }}{{\sum\nolimits_{i = 1}^{{N_{x} }} {\sum\nolimits_{j = 1}^{{N_{y} }} {(A_{i,j} - r_{i,j} )^{2} } } }}} \right),$$

where *N*_*x*_, *N*_*y*_ are the image dimensions, and *MAXI* is the maximum gray value of the image. *r*_*i,j*_ is the gray value of the phantom for pixel (*i*,*j*). In this study, images are all normalized to [0,1], so that *MAXI* equals to 1. Tables 1 and 2 list the PSNR values of three reconstruction algorithms. In all cases under study, the PSNR values provided of the DDTV are much better than those of FBP and TV. In Table 1 corresponding to the transverse texture, the PSNR of the DDTV is by about 16 an 10 dB higher than those of FBP and TV, respectively. As for the circular texture described in Table 2, the PSNR value of the DDTV is about 20 dB and 8.5 dB higher than those of FBP and TV, respectively. Even for the sparse-view sampling cases, the DDTV maintains its superiority. For the 30-view sampling of the circular texture presented in Table 2, the PSNR value of the DDTV surpasses those of FBP and TV by 20.58 and 12 dB, respectively. The PSNR results of PSNR comply with the visual quality analysis.

**Table 1:** PSNRs (dB) of the reconstructed transverse texture images

	180-view	90-view	30-view
FBP	16.12	14.57	13.13
TV	21.24	20.02	21.62
DDTV	32.06	32.29	28.41

**Table 2:** PSNRs (dB) of the reconstructed circular texture images

	180-view	90-view	30-view
FBP	12.96	11.23	9.19
TV	27.05	23.50	17.77
DDTV	31.43	32.17	29.77

**Fig. 5:**
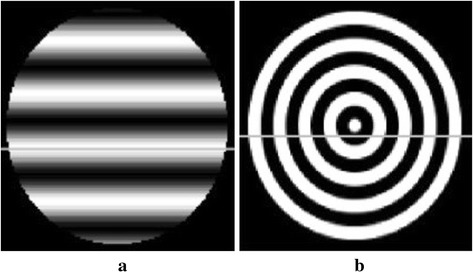
The location of the *pixel line* in the images for the transverse texture image (**a**) and the circular texture image (**b**)

**Fig. 6:**
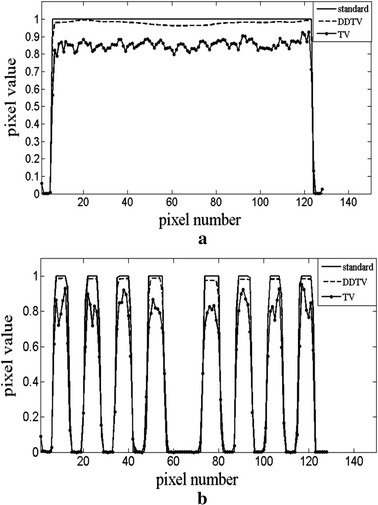
The *gray* value profiles of the DDTV and the TV reconstruction algorithm in comparison with the standard profile. **a** Is the profile for Fig. 5a and b is that of Fig. 5b

To display the details of the reconstructed images, a line of the pixel value of the 30-view reconstructed images for the two cases is taken out and compared with that of the original images. Figure 5a, b shows the location of the pixel line in the images, while Fig. 6a, b depicts the comparison curves of the pixel value for two images, respectively. The results obtained show that, as compared to TV, the DDTV profiles are much closer to the standard results, the range abilities of pixel values of the DDTV are much smaller in the homogenous region, and the variation trends coincide better with the true ones.

### Shepp–Logan phantom

**Fig. 7:**
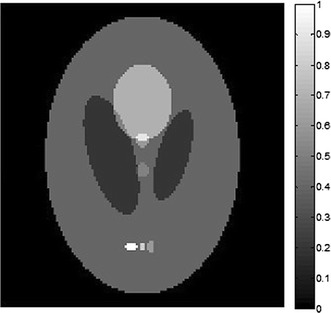
The Shepp–Logan phantom

**Fig. 8:**
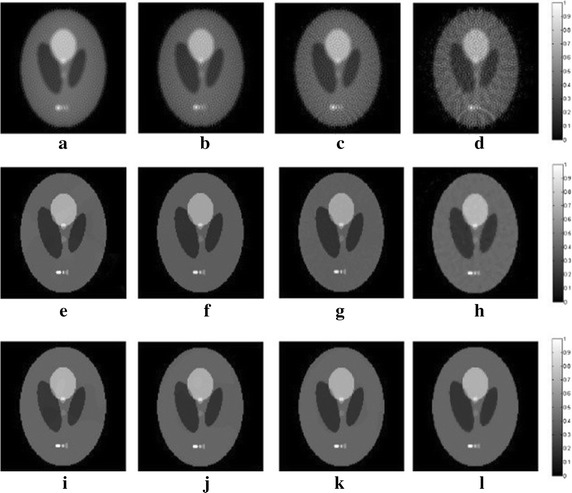
Reconstructed results of the Shepp–Logan phantom by the FBP, the TV and the DDTV, respectively. The first to third *rows* refer to reconstructed images by the FBP (**a**–**d**), the TV (**e**–**h**) and the DDTV (**i**–**l**), respectively. The first to fourth *columns* refer to results from 180-view (**a**, **e**, **i**), 90-view (**b**, **f**, **j**), 60-view (**c**, **g**, **k**), 30-view (**d**, **h**, **l**) sampling, respectively

**Fig. 9:**
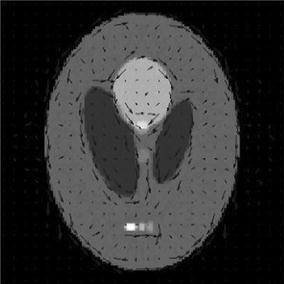
The orientation field estimation results in the last iteration of the DDTV from 30-view sampling. The *arrows* stand for the direction for the blocks. The length of the *arrows* indicates the reliability of the estimated orientation field for that position

The Shepp–Logan phantom is also adopted to further evaluate the effectiveness of the algorithm. The image domain dimensions and the scanning radius are the same as those as those in “Texture image reconstruction”. In this case, the angular interval of the sampling points are set to 2°, 4°, 6°, 12°, and the sampling points are 180, 90, 60, and 30, respectively. The parameters *λ* and *α*_*m*_ are set to 0.01 and 2.5, respectively. The Shepp–Logan phantom is shown in Fig. 7, and the reconstruction results of the DDTV, TV and FBP are shown in Fig. 8. The orientation field estimation results for the last iteration of the DDTV from 30-view sampling are displayed in Fig. 9. The arrow directions in Fig. 9 agree with the directivity pattern of the texture edges. In the isotropic parts of the image with weaker directivities, the arrow length is smaller than that in the edge parts with stronger directivities. Table 3 shows the comparison of the PSNR for the three algorithms.

**Table 3:** PSNRs (dB) of the reconstructed results of Shepp–Logan phantom

	180-view	90-view	60-view	30-view
FBP	15.35	15.36	15.24	14.68
TV	38.01	38.23	38.18	36.68
DDTV	44.97	41.60	40.37	37.78

When the number of sampling points is sufficient, all three algorithms provide excellent results, where each part of the phantom in the reconstructed images is distinguishable. However, there is a certain degree of blurs in the reconstructed images of the FBP, while the quality of FBP-reconstructed images sharply declines as the sampling points decrease with a lot of artifacts. The DDTV and the TV both have good performances for all sampling cases. However, the DDTV-reconstructed images have sharper image edges and clearer texture details, as compared to TV ones. When the number of sampling points is reduced to 30, the DDTV results exhibit no observable changes, while in the TV ones there appear some artifacts. Results of the PSNR listed in Table 3 provide a quantitative proof to the above findings: the PSNR values of DDTV are higher than those of FBP and TV by about 26 and 3.3 dB, respectively. For the sparse-view (i.e., 30-view) sampling, the PSNR provided by DDTV is by 23.1 dB higher than that of FBP and still exceeds that of TV by 1 dB.

### Multimode scanning

To test the validity of the DDTV in terms of multimode scanning, simulations are also conducted under the limited-view and linear scanning conditions. The same simulation environment and parameter settings as in the Shepp–Logan phantom are used. In case of limited-view scanning, the sampling interval is 4° and the number of sample points is equal to 30, which correspond to the sampling angle 120°. In case of linear scanning, the sampling length is 100 mm and the number of sampling points is 20.

**Fig. 10:**
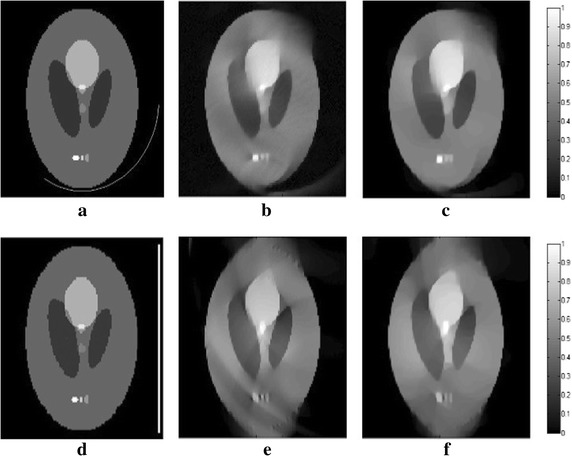
Reconstructed results of the TV and DDTV from limited-view and line-scanning respectively. **a**, **d** Refer to the scanning positions of limited-view and line-view samplings, respectively. **b**, **e** Refer to the reconstructed results of TV for two scanning modes, respectively. **c**, **f** Refer to the reconstructed results of DDTV for two scanning modes, respectively

The scanning diagram and the reconstruction images of two cases are shown in Fig. 10a–f. Results show that the DDTV can be applied for multimode scanning. But due to the deficiency of the information in some angles, some artifacts appear in both two reconstructed images. In the limited-view scanning results shown in Fig. 10b, there are certain blurs at the image edges, especially in outer contour of the top right corner. For the linear-view scanning results shown in Fig. 10d, images oriented in the horizontal direction are relatively good, but there are obvious blurs in the vertical direction of the image due to the less of information in that direction by linear-view scanning. The TV-reconstructed results for limited-view and linear-view scanning conditions are shown in Fig. 10b, e, respectively. Although there are some artifacts due to the lack of some angular information, DDTV exhibits better results, as compared to TV, in the case of limited-view scanning. Further improvements of the limited-view scanning results can be achieved by adding some compensation methods.

### Robustness of the algorithm

In practical experiments, the detected signals are readily interfered by the system noises, which are usually the white Gaussian ones, so it is necessary to analyze the noise robustness of the proposed algorithm.

**Fig. 11:**
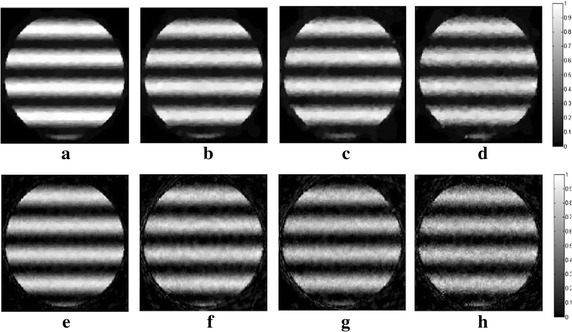
The first to second *rows* refer to the reconstructed results of the noise-added transverse texture images from the DDTV (**a**–**d**) and the TV (**e**–**h**), respectively. The SNR of the signals are 10 dB (**a**, **e**), 5 dB(**b**, **f**), 3 dB (**c**, **g**), and 0 dB (**d**, **h**)

**Fig. 12:**
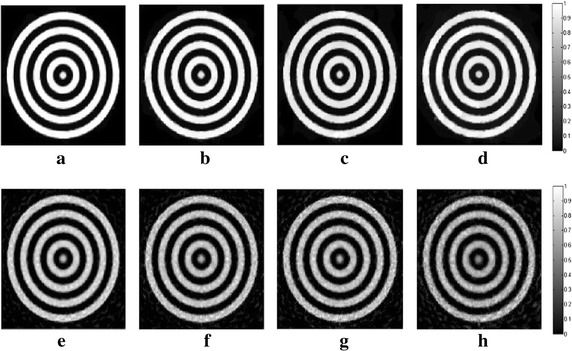
The first to second *rows* refer to the reconstructed results of the noise-added circular texture images from the DDTV (**a**–**d**) and the TV (**e**–**h**), respectively. The SNR of the signals are 10 dB (**a**, **e**), 5 dB (**b**, **f**), 3 dB (**c**, **g**), and 0 dB (**d**, **h**)

**Fig. 13:**
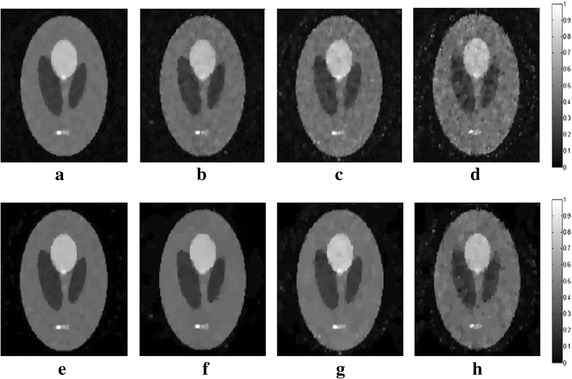
The first to second *rows* refer to the reconstructed results of the noise-added Shepp–Logan images from the TV (**a**–**d**) and the DDTV (**e**–**h**), respectively. The SNR of the signals are 10 dB (**a**, **e**), 5 dB (**b**, **f**), 3 dB (**c**, **g**), and 0 dB (**d**, **h**)

In this part of simulation studies, white Gaussian noises with the signal- to-noise ratio (SNR) values of 10, 5, 3, and 0 dB are added to the acquired photoacoustic signals in the case of 30-view scanning for texture images depicted in Fig. 1, as well as the Shepp–Logan image shown in Fig. 7. Results obtained via the TV and the DDTV methods are shown in Figs. 11, 12, and 13, while PSNR values are displayed in Tables 4, 5, and 6.

**Table 4:** PSNR (dB) of the noised-added transverse texture phantom

	10 dB	5 dB	3 dB	0 dB
TV	21.97	19.39	17.78	16.98
DDTV	28.24	25.66	24.05	21.83

**Table 5:** PSNR (dB) of the noised-added circular texture phantom

	10 dB	5 dB	3 dB	0 dB
TV	16.87	15.52	15.10	13.47
DDTV	26.98	24.57	22.13	21.37

**Table 6:** PSNR (dB) of the noised-added Shepp–logan phantom

	10 dB	5 dB	3 dB	0 dB
TV	32.24	28.01	22.44	16.96
DDTV	34.03	30.59	28.19	26.21

Both DDTV and TV methods succeed to maintain their effectiveness with high SNR. Taking the Shepp–Logan phantom as example, the respective results shown in Fig. 13; Table 6 reveal no strongly manifested influences on the reconstructed images, when SNR = 10 dB. The PSNR values of the DDTV and TV are excellent, amounting to 34.03 and 32.24 dB, respectively. However, the TV performances deteriorate distinctly as the SNR decreases. Especially, when the SNR drops to 0 dB, the reconstructed image of the TV is affected significantly. A lot of background noises can be observed, and the texture details are blurred in the TV outputs, as is shown in Fig. 13d. The PSNR values of the TV decline from 32.24 for 10 dB to 16.96 for 0 dB. On the contrary, the DDTV demonstrates the superiority of noise robustness over the TV. It provides high image quality even under the condition of low SNR. The PSNR of the DDTV for 3 dB SNR is by 5.75 dB higher than that of the TV. For the case of 0 dB SNR, the PSNR of the DDTV is by 9.25 dB higher than that of the TV.

### Convergence and calculation

The convergence speeds of two iteration algorithms are compared by the distance between the reconstructed and original images, which is defined as:

25 $$d = \left( {\frac{{\sum\nolimits_{i = 1}^{{N_{x} }} {\sum\nolimits_{j = 1}^{{N_{y} }} {\left( {A_{i,j} - r_{i,j} } \right)^{2} } } }}{{\sum\nolimits_{i = 1}^{{N_{x} }} {\sum\nolimits_{j = 1}^{{N_{y} }} {r_{i,j}^{2} } } }}} \right)^{1/2} ,$$

where *d* expresses the degree of difference between the reconstructed and original standard images. The smaller *d*, the less difference with the original image.

**Fig. 14:**
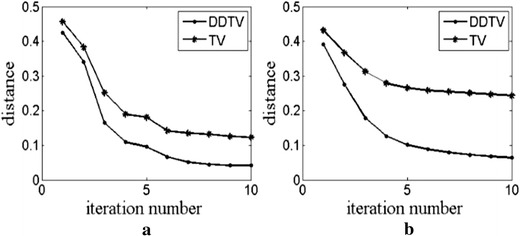
The distance between the reconstructed image and the original image versus the iteration number. **a** Is for 90-view simulation and **b** is for 30-view simulation

We record *d* of two methods for each iteration step in cases of 30- and 90-view circular texture image reconstructions. The chart of *d*-iteration step number is shown in Fig. 14a, b. One can see that the value of *d* for the DDTV is smaller than that of the TV for each iteration step in both two cases, while the differences of *d* between two algorithms enlarge as the iteration number increases. For 90-view scanning shown in Fig. 14a, the values of *d* for the DDTV decrease from 0.43 to 0.04 within 10 iteration steps, as compared to variation from 0.46 to 0.12 observed for the TV. For 30-view scanning shown in Fig. 14b, the values of *d* for the DDTV decrease from 0.39 to 0.06 within 10 iteration steps, as compared to the drop from 0.43 to 0.24 of the TV. The results obtained strongly indicate that the DDTV is more accurate and has a faster convergence speed than the TV.

Although TV and DDTV may not converge to the original image, the ultimate goal of using these two algorithms is to reconstruct images, as close as possible to the true ones. The results depicted in Fig. 14 show that as the iteration goes, the distance between the reconstructed and original images decreases within 10 iterations for both algorithms. The distance to iteration curve converges gradually, which validates the convergence of these algorithms. Although the algorithms may only converge to the suboptimal solutions, which approach the optimal one, Eq. (25) still holds for indication of the algorithm convergence property by taking the true images as the gold standard. The distance *d* in (25) has also been used as an indication of the algorithm convergence in [31] and [47]. Researchers [21–25] also used true images as a standard to calculate the root-mean-square error for each iteration to study the convergence of the iterative PAT-reconstructed algorithms.

The computation time is another indicator, which needs to be considered for the evaluation of iterative algorithms. Although TV and DDTV have different optimization algorithms, the proposed DDTV is not a fast algorithm, intended to improve the operation speed. So we compare the computation costs for one iteration step between the TV and the DDTV to show that DDTV has more efficient computation than TV. It is found that the computation times of one iteration step for the TV and the DDTV are roughly the same. For example, the average calculation times of one iteration step for 30-view circular scan for a 128 × 128 pixels image are 1.5990 and 1.575 s for the TV and the DDTV, respectively. For 90-view, the respective times are 4.257 and 4.366 s for the TV and the DDTV, respectively. Insofar as DDTV has a higher convergence speed than TV, and the computation costs for one iteration are close for the two algorithms, it outperforms the TV according to both factors.

### Universality

**Fig. 15:**
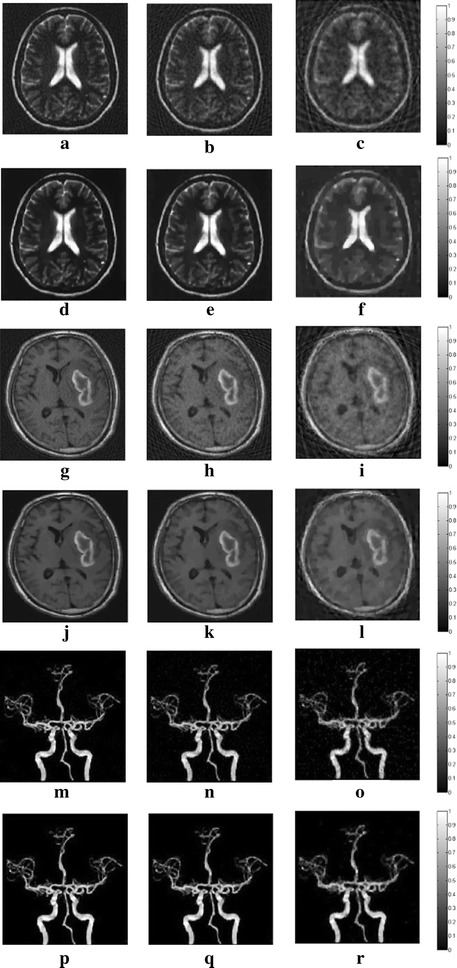
The reconstructed images of three different medical images from the TV and the DDTV. The first, third and fifth *rows* refer to the results from the TV (**a**–**c**, **g**–**i**, **m**–**o**). The second, fourth and sixth *rows* refer to that of the DDTV (**d**–**f**, **j**–**l**, **p**–**r**). The first to third *columns* refer to 90-view (**a**, **d**, **g**, **j**, **m**, **p**), 60-view (**b**, **e**, **h**, **k**, **n**, **q**), 30-view (**c**, **f**, **i**, **l**, **o**, **r**) simulations, respectively

To test the universality of the algorithm, we also choose three medical images as the original optical absorption distribution, which are two MRI brain images and one angiography image. The reconstruction results of the DDTV and the TV are displayed in Fig. 15. For the MRI brain images, the reconstructed images of the DDTV, as is shown in Fig. 15d–f, i–l, have more distinct image edges and texture details. Moreover, the central paracele and sulci in the first brain image have a higher contrast and the tumor in the second brain image is clearer for the DDTV than that of the TV, as is shown in Fig. 15a–c, g–i. As for the angiography image, one can observe that DDTV images shown in Fig. 15p–r have better performances in terms of image contrast and texture detail preservation. These results confirm the DDTV applicability for various kinds of images.

## Experimental results

This Section briefly describes the in vitro experiments conducted to verify the DDTV algorithm. We first made two vessel-like phantoms to test the effectiveness of the proposed DDTV algorithm through 90- and 30-view circular scanning. The reconstructed results of the DDTV were compared with those of FBP and TV. Linear scanning experiments were also conducted, with the DDTV and TV results being compared and discussed.

**Fig. 16:**
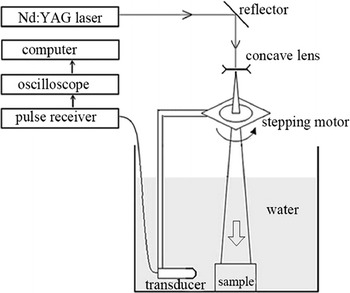
The experimental platform scheme

The experimental platform is shown in Fig. 16. A laser beam irradiated with a Nd:YAG laser device (Contimuum, Surelite I) is reflected by a mirror and then transferred through a concave lens. The wavelength of the laser in this experiment is 532 nm. The duration and repetition frequency of the laser pulse are 4–6 ns and 10 Hz, respectively, which comply with the experimental requirements. The phantom is homogeneously illuminated from above, and photoacoustic signals are detected from the side. The transducer (V383-SU, Panametrics) is unfocused with the center frequency of 3.5 MHz and bandwidth of 1.12 MHz. A stepping motor drives the transducer to scan around the phantom. The sampling frequency of the system is 16.67 MHz.

**Fig. 17:**
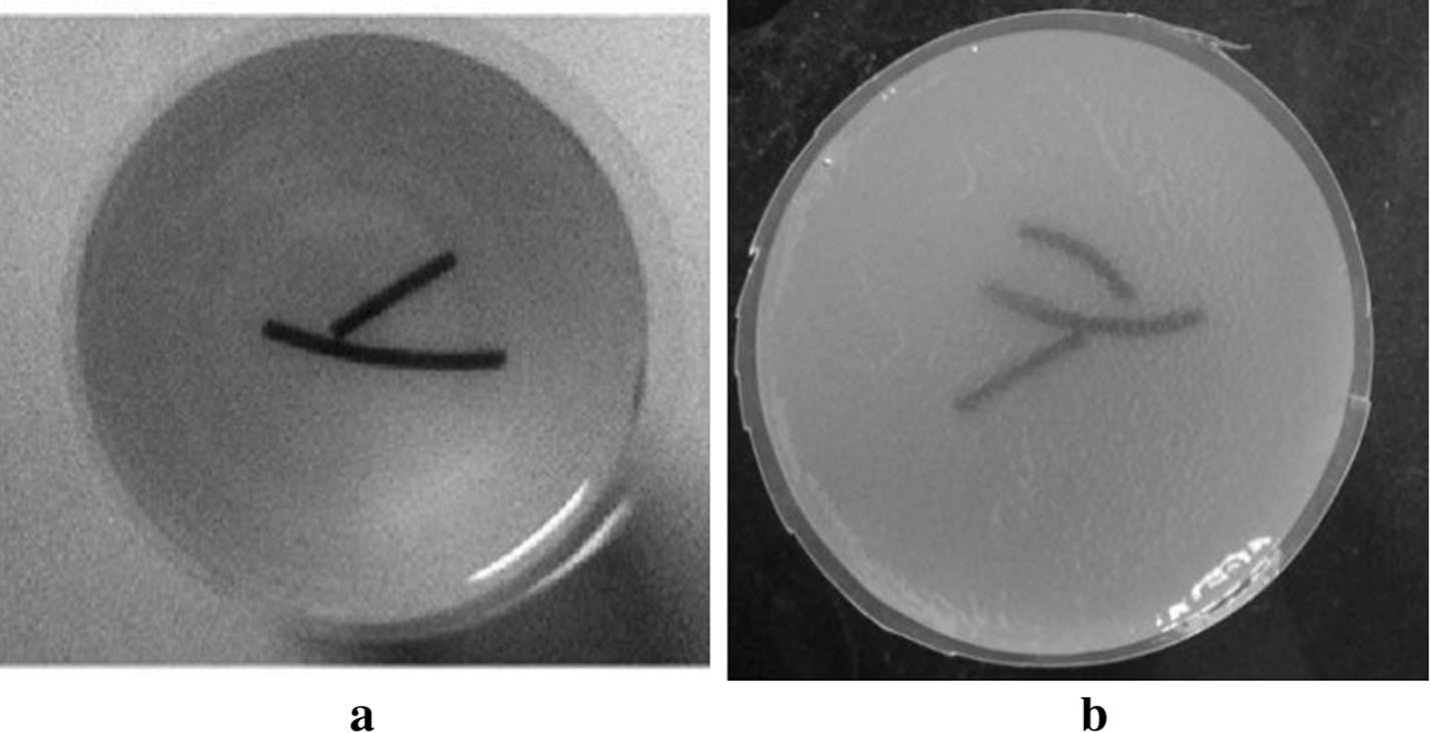
The picture of the circular scan experiment phantom

The experiments on the phantoms (Fig. 17) have been earlier performed by the authors and reported in [31, 47]. These experimental data are referred to in this study, in order to verify whether the proposed algorithm has a better performance than the TV-based one under the same test conditions with the same experimental data. Also, we added a new linear scan experiment in Figs. 20 and 21 to show that our method is capable of accurate image reconstruction under different kinds of scanning modes.

The phantoms used in Fig. 17 are made of gelatin with the tissues to be imaged embedded into the gelatin cylinder. The phantom shown in Fig. 17a uses two rubber bars of 20 and 12 mm in length, respectively. As is shown in Fig. 17b, three wires with the diameter of 1 mm each are embedded into the gelatin. The diameters of both phantoms are 50 mm. Transducer scans around the phantom circularly with the scanning radius of 38 mm. The angular intervals of the sampling points are set to 4° and 12°, which corresponds to 90- and 30-view samplings, respectively. The laser energy density is set to meet the ANSI laser radiation safety standards.

**Fig. 18:**
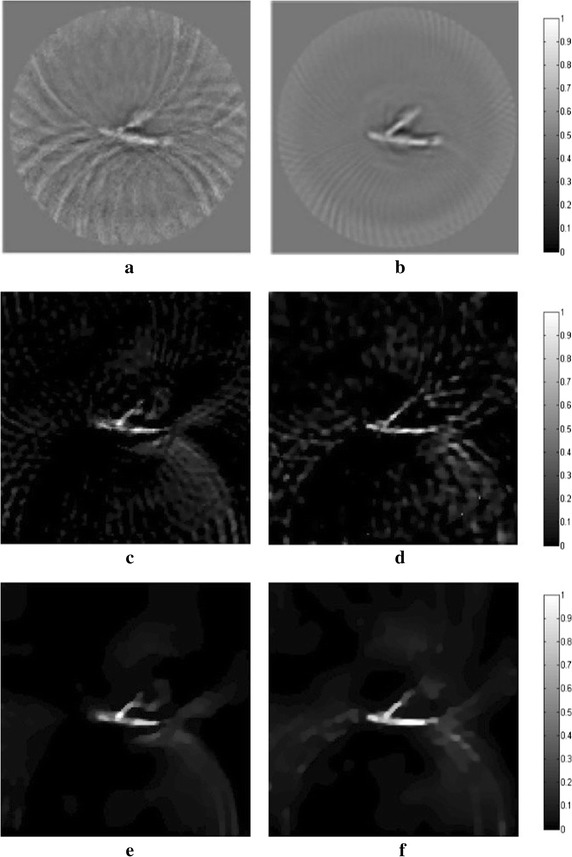
The reconstructed images of the phantom in Fig. 15a from 30-view (**a**, **c**, **e**) and 90-view (**b**, **d**, **f**) detected *points*, respectively. The first to third *rows* refer to results from the FBP (**a**, **b**), the TV (**c**, **d**) and the DDTV (**e**, **f**), respectively

**Fig. 19:**
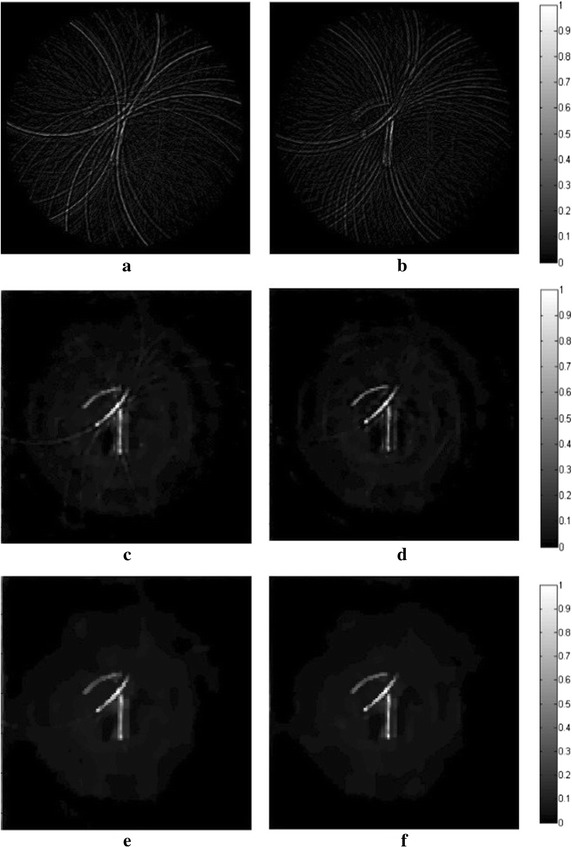
The reconstructed images of the phantom in Fig. 15b from 30-view (**a**, **c**, **e**) and 90-view (**b**, **d**, **f**) detected *points*, respectively. The first to third *rows* refer to results from the FBP (**a**, **b**), the TV (**c**, **d**) and the DDTV (**e**, **f**), respectively

The experimental reconstruction results obtained by FBP, TV, and DDTV are shown in Figs. 18 and 19 and confirm that all three algorithms can provide nearly perfect reconstruction results, when the number of sampling points is sufficient. However, for 30-view sampling, the image quality of the FBP declines sharply, with appearance of multiple artifacts. Both TV and DDTV maintain their effectiveness in sparse-view sampling. As compared to the TV, the DDTV shows its advantages in terms of the reconstruction results. The contrast of the images is enhanced and the edges of the image are more distinct. So the DDTV is preferable to TV for maintaining the image texture details.

**Fig. 20:**
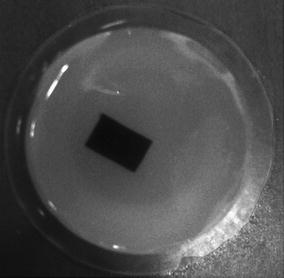
The picture of the linear scan experiment phantom

We also performed the linear scanning experiments. The phantom is also a gelatin cylinder with the diameter of 50 mm, which is embedded into a rectangular rubber slice acting as the laser beam absorber. The slice dimensions are 9 mm × 14 mm. The phantom is shown in Fig. 20. The photoacoustic signals are sampled uniformly aligning to the longer edge of the rectangle. The sampling interval is 1 mm and the number of the sampling points is 41.

**Fig. 21:**
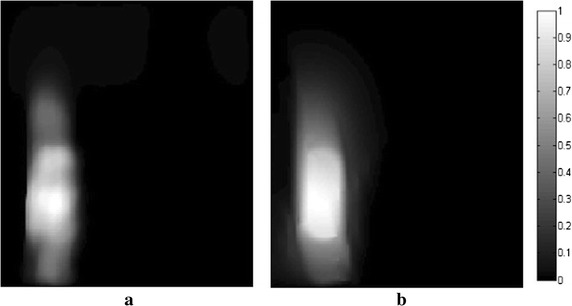
The reconstructed image of the linear scan experiment from the TV (**a**) and the DDTV (**b**), respectively

The results of the TV and the DDTV are shown in Fig. 21. Due to the incompleteness of the angular information of the linear scan, platelike artifacts appear in both TV and DDTV results. But the DDTV shows sharper edges of the laser beam absorber. The profile of the rectangle is clearly visible and the pixel values are distributed more uniformly within the rectangle. So the DDTV is more effective than the TV for the linear scan experiments.

## Discussion and conclusion

In this study, a novel model-based photoacoustic image reconstruction algorithm using the directivity adaptive direction total variation (DDTV) is proposed to minimize the deficiencies of the PAT image reconstruction algorithms in terms of image edges and texture details preservation. The classical TV is a sum of L_2_ gradient norms of the image, which measures the variations and penalizes local changes in the image regardless of the image directions. So the TV-based algorithms perceive that the reconstructed images are piecewise-constant, which may hold for cartoon-like piecewise images, while most natural image reconstructions violate this assumption. The TV models in such applications as image denoising problems minimize the TV along all directions, which leads to a disredard of the important directivity information of images. Therefore, classical TVs are not suitable for images with strong directivity patterns. When applied to PAT image reconstruction methods, the TV-based algorithms tend to produce over-smoothed image edges and texture details, since the TV assumption favors piecewise-constant solutions. So the problem of TV-based PAT reconstruction is that it fails to obtain adequate results, in terms of texture detail preservation, which is critical for PAT images with spatially varying directivity patterns, such as blood vessel imaging, tumor detection, biological tissue microstructures analysis. The classical TV-based PAT reconstruction methods fail to provide ideal directional information in terms of the reconstructed results. So the application of DDTV to PAT is quite lucrative, since it calculates the TV based on the spatially varying directivity patterns of the image, which makes the TV sensitive to the image directions. The anisotropic directivity pattern of the image is estimated adaptively during the iterations, and the image gradients are weighted by the estimated orientation fields, which makes is applicable to all kinds of images with various directivity patterns. Two kinds of parameters are calculated for the orientation field estimation: direction *θ* and reliability of the estimated direction *C*_*k*_, which control the TV directions and the sensitive degree for those directions, respectively. This dual-weight method assures the accuracy of the DDTV parameter for the image.

The maximum major axis *α*_*m*_ defines the largest weight of TV in the chosen direction *θ*_*k*_. When the orientation pattern is strong, the weight of TV in that direction reaches *α*_*m*_. Small values of *α*_*m*_ may lead to worse performance of DDTV as to preserving the edges and texture details of the image, in comparison to that of TV. In a more extreme case, when *α*_*m*_ equals unity, the ellipse turns into a directionless ball, which makes DDTV equal to TV. Vice versa, too large values of *α*_*m*_ will cause the oversized weight of TV in *θ*_*k*_, leading to a relatively small TV in that direction. Thus, the reconstructed image will be distorted. Therefore, *α*_*m*_ should be set to a value which is neither too small nor too large. In general, for images with stronger direction patterns *α*_*m*_ should be larger, while for relatively homogeneous ones, it should be smaller. In the simulations, the phantoms of texture images have relatively strong direction patterns, so that *α*_*m*_ = 10 is used in this case to obtain the best performance. The Shepp–Logan phantom is comparatively uniform with a weak directivity, so *α*_*m*_ in this case is set to 2.5. The simulations of this study can be used as a reference for selection of *α*_*m*_, while in general reconstruction cases *α*_*m*_ can be set between 2–10.

The DDTV was implemented into the model-based PAT reconstructed algorithm, and the primal–dual based method described in [43] is utilized to solve the optimization problem iteratively. Numerical simulations and in vitro experiments verify the effectiveness of the algorithm. The reconstructed images are studied qualitatively and quantitatively. Simulation results show that the DDTV method has a better performance than FBP and TV, in terms of PSNR, robustness, convergence speed, and visual quality, while its superiority is exhibited even for sparse-view and multi-mode sampling cases. As reported in [31], the TV algorithm converges when the number of iterations is 10, which number is a relatively appropriate choice for TV algorithms. The experimental data show that DDTV also converges about 10th iteration, so the average converging number of 10 was also selected. As shown in “Convergence and calculation”, the line chart of the distance *d* in Fig. 14 also confirms that the distance between the iteration curves converges when the number of the iterations is 10, which validates the convergence of the algorithms at 10th iteration. From simulations of various images, it can be observed that the dominants of the proposed DDTV algorithm are more prominent for images with a stronger directivity. For the two texture images, chosen in the first part of simulation, the values of PSNR of DDTV are by 16.24 and 9.96 dB higher than those of FBP and TV, respectively, for the transverse texture image. For the circular image, the respective excesses are 19.99 and 8.35 dB over those of FBP and TV. Other texture images with different texture patterns were also tested, with the PSNR values of DDTV exceeding those of FBP and TV by about 18 and 10 dB for all the cases. As for the Shepp–Logan phantom in the second part of simulation, the superiority of DDTV is less pronounced as that of texture images, because the directivity of the Shepp–Logan is not so strong and the major part of the image corresponds to smooth areas with no directions. So, for images a with weak directivity, the performances of DDTV and TV are similar, but the improved quality of the reconstructed images is also confirmed by the Shepp–Logan phantom results. Due to different mechanisms for the reconstructed algorithms, the gray level of the reconstructed images would be different. There may be a certain degree of amplification or narrowing for the gray level of the reconstructed images, as compared to the original ones. Therefore, it is difficult to compare the results for those algorithms under different gray levels. The images are normalized via dividing the gray values of all pixels by the maximum gray value of the images, which has no effect on the quality of images. Also when comparing the PSNR and *d* for all algorithms, the reconstructed images and the original images should be at the same gray level. Therefore, the images in this study are normalized in the same gray level for comparison. The simulation results also suggest although the DDTV has a higher convergence speed, than that of the TV, it fails to improve the calculation time. The calculation times for an iteration step of the DDTV and the TV are basically the same. Thus, further studies are needed to develop a faster and more efficient method to solve the iterated problem. Moreover, the reconstructed result of the DDTV in the linear-view scanning is no better than those obtained via circular and limited-view ones. The information in the vertical direction of the image is not properly reconstructed. So we also need to modify the proposed algorithm to improve the performance for the linear scanning in the future.

In-vitro experiments further prove that the proposed DDTV algorithm is able to reconstruct images with a higher quality than FBP and TV algorithms for both circular and linear scanning cases. The contrast of the images is enhanced and the edges of the image are more distinct. The profiles of the optical absorbers are clearer and the texture details information of the images are easier to observe. Thus, the DDTV-based PAT reconstruction algorithm has very promising prospects for biomedical applications, especially those, which need the texture detail information of the tissue. In the experiments, simple gelatin-based phantoms are used. A single, low-frequency transducer is used in the experiment to detect the PAT signals by scanning around the phantom, which is quite time-consuming and inconvenient for practical application. In the future, we will improve the experimental system for more complicated biological tissue and in vivo experiments. Consequently, the results of numerical simulations and experiments corroborate that the proposed DDTV is an effective algorithm for the PAT image reconstruction, which has a number of advantages over the available FBP and classical TV-based algorithms.

## Abbreviations

PA: photoacoustic
PAT: photoacoustic imaging
TV: total variation
DDTV: directional total variation with adaptive directivity
ADTV: adaptive directional total-variation
PSNR: peak signal-to-noise rations
FBP: filtered back-projection method
CS: compressed sensing
ASD-POCS: adaptive steepest-decent-projection onto convex sets
DTV: directional total variation
FDTD: finite-difference time-domain
SNR: signal-to-noise ratio
